# Maternal congenital heart disease and risk of child developmental vulnerability in early school age: A population-based cohort study

**DOI:** 10.1371/journal.pmed.1004890

**Published:** 2026-07-02

**Authors:** Muhammad Zakir Hossin, Anne Gadermann, Edit Nagy, Randip Gill, Monique Gagné Petteni, Jonas Faxén, Neda Razaz

**Affiliations:** 1 Division of Clinical Epidemiology, Department of Medicine, Solna, Karolinska Institutet, Stockholm, Sweden; 2 Human Early Learning Partnership, School of Population and Public Health, University of British Columbia, Vancouver, Canada; 3 Department of Cardiology, Karolinska University Hospital, Stockholm, Sweden; 4 Department of Physiology and Pharmacology, Karolinska Institutet, Stockholm, Sweden; Stellenbosch University, SOUTH AFRICA

## Abstract

**Background:**

While maternal congenital heart disease (CHD) is associated with increased risks of adverse pregnancy outcomes, its impact on long-term child development remains unknown. This study aimed to investigate if in-utero exposure to maternal CHD is associated with child developmental vulnerability at school entry.

**Methods and findings:**

This population-based cohort study included 256,629 singleton offspring born in British Columbia, Canada between January 1, 1995 and December 31, 2016, with follow up through linkage to teacher-rated Early Development Instrument (EDI) surveys administered in kindergarten around 5–6 years of age. Over 90% children enrolled in participating schools completed the questionnaire. Developmental vulnerability was defined as a score <10th percentile in any two of the five EDI domains: physical health and wellbeing, social competence, emotional maturity, language and cognitive development, and communication and general knowledge. The association between maternal CHD and child developmental vulnerability was examined using modified Poisson regression models, adjusted for maternal age at delivery, parity, country of birth, marital status, neighborhood income quintiles, preexisting psychiatric disorders, and pre-gestational diabetes. A counterfactual four-way decomposition method was used to quantify potential mediation and moderation by preterm birth. Of the 256,629 children (51.4% female) included in the analysis, 456 (0.2%) were exposed to maternal CHD. Developmental vulnerability was identified among 25.2% children exposed to maternal CHD compared with 16.6% among the unexposed. In the adjusted model, maternal CHD was associated with 28% higher risk of developmental vulnerability (aRR 1.28; 95% CI [1.11, 1.48]) compared with no maternal CHD. The increased risk was observed across multiple developmental domains related to physical health and wellbeing (aRR 1.31; 95% CI [1.11, 1.54]), social competence (aRR 1.22; 95% CI [1.02, 1.45]), language and cognitive development (aRR 1.39; 95% CI [1.13, 1.70]), and communication and general knowledge (aRR 1.33; 95% CI [1.09, 1.63]). Preterm birth mediated only about 8% of the overall association. Severe CHD was more strongly associated with developmental vulnerability (aRR 1.98; 95% CI [1.31, 3.00]) compared to mild CHD (aRR 1.19; 95% CI [1.00, 1.42]). However, the study had limited capacity to separate intrauterine effects from potential genetic and postnatal familial influences. Some degree of CHD misclassification is possible, which would likely bias the association toward the null.

**Conclusions:**

In this population-based study, maternal CHD was associated with child developmental vulnerability at school entry. While further research is required to elucidate the mechanisms, enhanced clinical monitoring and tailored support to reproductive age women with CHD may help reduce the risk of developmental vulnerability in their children.

## Author summary

**Why was this study done?:**

- With advances in cardiac care, most women born with congenital heart disease (CHD) survive to reproductive age and many become pregnant.
- While short-term pregnancy and birth outcomes for women with CHD are increasingly documented, little is known about the long-term health and developmental outcomes of their children.
- Understanding whether maternal CHD is associated with developmental vulnerability in offspring is important for prenatal counseling, perinatal care, and early intervention.

**What did the researchers do and find?:**

- We conducted a large population-based cohort study using linked health and early education data to assess the association between maternal exposure to CHD and child developmental outcomes.
- We assessed child developmental outcomes of 256,629 males and females at school entry, using a well-validated, population-level measure covering multiple developmental domains and subdomains relevant to health, learning, and social functioning.
- Maternal CHD was associated with a higher risk of developmental vulnerability compared with no maternal CHD, with consistent associations observed across physical, social, communication, and cognitive domains.
- The increased risk was not adequately mediated by preterm birth.

**What do these findings mean?:**

- As the population of women with CHD entering pregnancy grows worldwide, our findings highlight the importance of recognizing maternal CHD as a long-term risk factor for child development.
- The findings underscore the need for optimizing preconception and perinatal care for women with CHD, alongside early developmental monitoring for their children.
- However, maternal CHD may have been underdiagnosed and the study had limited capacity to distinguish pregnancy-related mechanisms from shared genetic influences, warranting further research.

## Abstract 3

In this population-based cohort study, Muhammad Hossin and colleagues assess developmental outcomes in children to assess whether maternal congenital heart disease is linked to increased risk of developmental vulnerability in their offspring.

## Introduction

Congenital Heart Disease (CHD) is the most common group of birth defects that affect nearly 1% of all live births. In 2019, an estimated 13.3 million individuals worldwide were living with this condition [1]. With major advances in pediatric cardiac care and surgical interventions, more than 90% of children born with CHD are surviving into adulthood [2,3], leading to a rapidly growing pregnant population with preexisting CHD [4–7]. The increased metabolic demands of pregnancy place extra strain on the maternal cardiovascular system [8], which may be poorly tolerated in CHD patients, especially among those with defects of moderate or severe complexity [6,9].

Epidemiological studies have shown that pregnancies complicated by preexisting CHD are associated with a higher risk of adverse obstetric and neonatal outcomes compared with the general pregnant population [10–13]. In particular, women with CHD have an increased risk of preterm birth and small-for-gestational age (SGA) birth [11], both of which are established risk factors for subsequent neurodevelopmental impairment, including motor delays, cognitive deficits, and poorer school performance [14,15]. Although the pathophysiological mechanisms remain unclear, the adverse pregnancy and neonatal outcomes in the CHD population are thought to be partly driven by impaired placental function [16]. Emerging evidence suggests a relationship between maternal cardiac dysfunction and placental insufficiency [16–18], which may hamper delivery of nutrients and oxygen to the fetus and adversely impact fetal nutrition, growth, and development [17,19].

While previous studies aiming to examine the in-utero effects of maternal CHD primarily focused on immediate perinatal outcomes, the long-term consequences beyond birth or infancy remain mostly overlooked [20]. Despite growing evidence of abnormal placental function and increased frequency of perinatal complications in mothers with preexisting CHD [17,21], it remains unknown whether children born to these mothers experience developmental difficulties during childhood. Therefore, in this study, we aimed to address this evidence gap by investigating whether intrauterine exposure to maternal CHD is associated with an increased risk of child developmental vulnerability at school entry. Additionally, we aimed to quantify the extent to which the estimated association is mediated by key neonatal conditions including preterm birth and SGA.

## Methods

### Data sources

Data for this population-based cohort study were primarily obtained from the British Columbia (BC) Vital Events and Statistics—Births database, which has recorded nearly all births in the province since 1985 [22]. This database contains information on maternal characteristics, medical conditions, pregnancy and delivery complications, and neonatal complications. These records were linked to several BC population-based databases: the Discharge Abstract Database (DAD, inpatient care) [23], the Medical Services Plan (MSP) physician billing data (primary and specialized outpatient care) [24], PharmaNet (medication dispensation) [25], Canadian Census data (for neighborhood income data), the Perinatal Data Registry [26], the Central Demographics File [27], and the Early Development Instrument (EDI) survey data (from 1999/2000 to 2022) [28]. Linkage across these databases was performed by Population Data BC using individual-level identifiers.

### Study population

**Fig 1:**
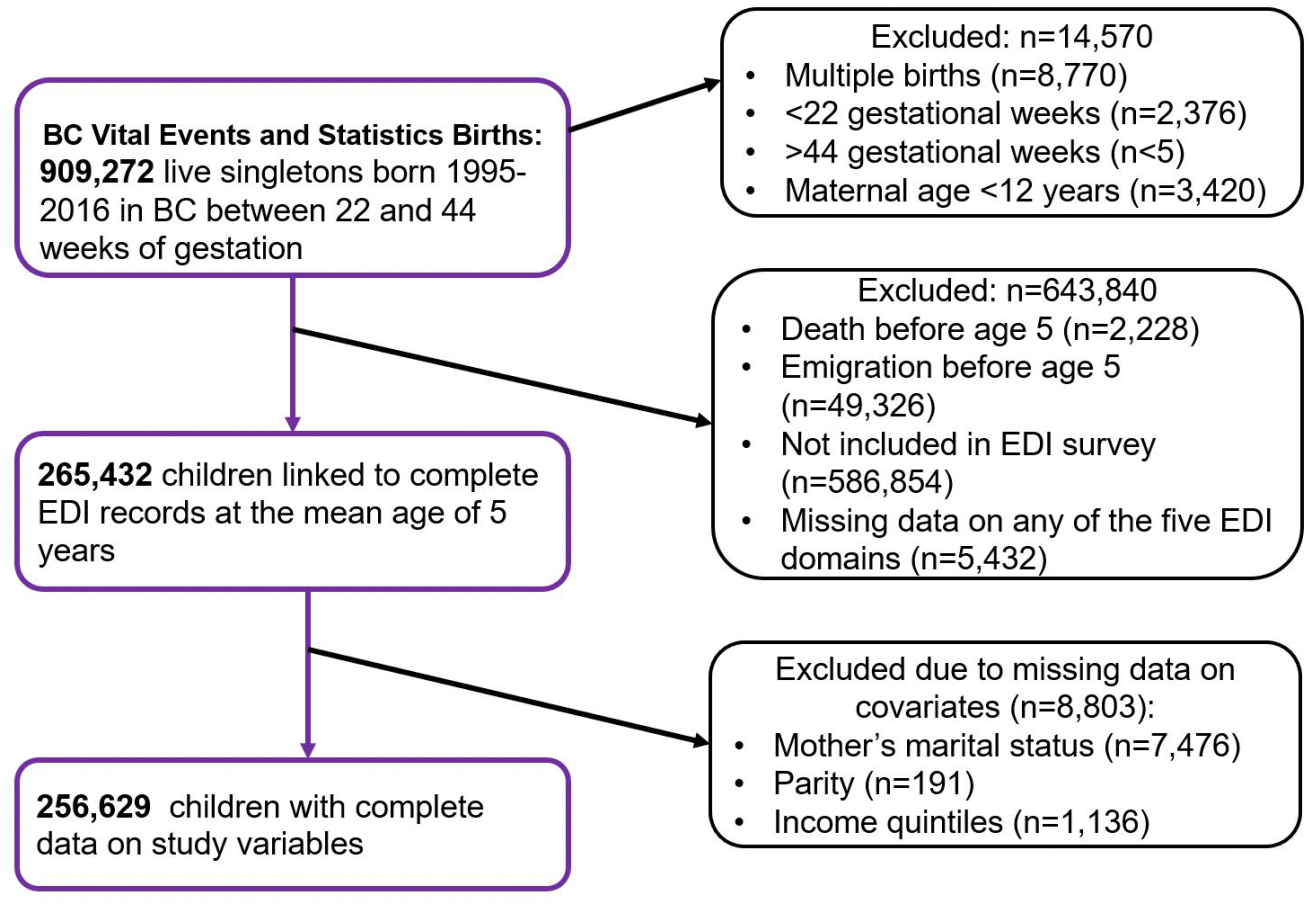
Flow diagram of the study sample. Note: BC, British Columbia; EDI, Early Development Instrument.

In the Vital Events and Statistics—Births database, we identified 909,272 singletons live-born between 22 and 44 completed weeks of gestation in BC, Canada from January 1, 1995 to December 31, 2016. These birth cohorts were included to coincide with reaching kindergarten age (5–6 years) at the time of EDI assessment between 1999 and 2022. The eligible study cohort consists of 265,432 children who had EDI records in kindergarten. Of them, 256,629 (96.7%) children had complete data on the key study variables and were included in the final analytic sample (Fig 1).

### Measures

#### Exposure.

Children were considered exposed in-utero if their mothers ever received a CHD diagnosis prior to conception. Maternal CHD was identified using the International Classification of Disease (ICD-9) and ICD-10 codes (Table A in S1 Appendix), based on at least one record in the BC inpatient or outpatient database. To improve diagnostic accuracy, CHD diagnoses from the MSP records were restricted to those with an associated fee item for cardiology, indicating that the diagnosis was made by a cardiologist. The primary exposure was any type of maternal CHD. In addition, we categorized CHD cases by level of complexity into mild, moderate, and severe CHD, based on the established classification system as detailed in the guidelines of European Society of Cardiology and American Heart Association/American College of Cardiology [29,30]. In BC, both MSP and DAD are part of a universal healthcare system, with MSP covering all outpatient fee-for-service physician encounters, while the DAD captures all hospitalizations and day surgeries with high coding accuracy [31]. The DAD switched from ICD-9 to ICD-10 coding in 2001 [32], whereas the MSP continued to use ICD-9 codes throughout the study period. Validation studies demonstrated that ICD-codes can reliably identify patients with CHD, with excellent sensitivity and reasonable specificity, although finer categorization by specific defects or disease severity may be less robust [33,34].

#### Outcomes.

The main outcome was child developmental vulnerability assessed through the EDI questionnaire, a population-level developmental monitoring instrument administered in kindergarten in public and participating independent schools across nearly all BC districts [28,35]. The EDI is a 103-item questionnaire completed by the school teachers in the classroom during the latter half of the kindergarten year. Following the pilot EDI survey conducted in 1999/2000, data have been routinely collected in systematic multi-year waves across the province, with each wave typically spanning three years. On average, more than 90% children enrolled in participating schools and school districts completed the questionnaire (Table B in S1 Appendix).

The EDI was designed to evaluate children’s competencies across five core domains of early development needed for effective participation in school-based learning. These include physical health and wellbeing (13 items), social competence (26 items), emotional maturity (30 items), language and cognitive development (26 items), and communication skills and general knowledge (8 items). The EDI items included multiple choice response options, of which the teachers selected the option that best described the student over the past 6 months. Each item was rated on either a 2-point or a 3-point Likert scale, with assigned scores of 0/10 or 0/5/10, respectively. An additional “don’t know” response option was available and was coded as missing. All domains of child development, except communication skills and general knowledge, comprise 3–4 subdomains each assessing specific skills and behaviors [28]. Each domain was standardized to calculate the same minimum (0 = low ability) and maximum scores (10 = high ability). Although the pilot and wave 1 surveys initially used more response categories, the EDI items themselves and the standardization procedure remained consistent across all survey waves.

The primary outcome was developmental vulnerability defined as scoring below the lowest 10th percentile on two or more EDI domains, based on a BC provincial cutoff derived from a normed sample [36,37]. While the EDI is not a diagnostic tool, the 10th percentile cutoff was partly chosen to identify children experiencing developmental difficulties that might not be clearly visible, rather than limiting the definition to those who could be clinically diagnosed. The rationale and detailed methodology of the percentile-based definition of developmental vulnerability were described elsewhere [36]. The EDI instrument showed high reliability and validity and was found to be predictive of later health, academic achievement, and other social outcomes [35]. The instrument also demonstrated good psychometric properties in several other countries [38].

In addition to overall vulnerability, we analyzed the EDI domains and subdomains as separate outcomes. Each subdomain was dichotomized to indicate if a child was “ready” or “not ready” for school, based on a criterion-referenced fixed threshold established by the EDI expert team at the Offord Centre [39]. We also examined severity of vulnerability as a cumulative risk of vulnerability across multiple developmental domains.

#### Mediators.

The two potential mediators selected a priori based on existing literature were SGA and preterm birth [11,14,15]. Preterm birth was defined as delivery before 37 completed weeks of gestation, with gestational age assigned based primarily on ultrasound before 20 weeks or the last menstrual period. SGA was defined as birth weight below the 10th percentile for gestational age and sex, using BC-specific growth reference curves [40].

#### Covariates.

The covariates examined in the study were maternal and child characteristics. Child characteristics included sex, birth year, and age at EDI completion, major congenital malformations diagnosed within the first year of life, and child CHD ever diagnosed after birth. Maternal characteristics were age at delivery, parity, country of birth, marital status, neighborhood income quintiles, preexisting psychiatric disorders, pre-gestational diabetes, gestational diabetes, cardiac medication use during pregnancy, and ischemic placental disease. Ischemic placental disease was defined as a composite of three obstetric complications—preeclampsia, placental abruption, and delivery of an SGA infant—which may share a common pathophysiological pathway involving poor placental function [41].

Maternal socio-demographic and birth characteristics were primarily obtained from the Vital Events and Statistics—Births database, whereas the disease covariates were derived from the inpatient, outpatient, and Perinatal Data Registry (Table C in S1 Appendix). Neighborhood income quintiles (from the Central Demographics File) were assigned using Statistics Canada’s Postal Code Conversion File (PCCF+) software, which links individual’s 6-digit postal codes to census-based income information at the dissemination area level [42]. Data on maternal medication use (i.e., beta blockers and calcium channel blockers) were retrieved from BC PharmaNet, using Anatomical Therapeutic Chemical (ATC) codes (Table C in S1 Appendix).

### Statistical analyses

All analyses were carried out in Stata version 16.0 (S2 Code). Maternal and child characteristics were compared between women with and without CHD using descriptive statistics. Modified Poisson regression models with robust (sandwich) variance estimation [43] were fitted to estimate the Risk Ratios (RR) and 95% Confidence Intervals (CI) for the association between maternal CHD and child developmental vulnerability. This approach is preferred over logistic regression when the outcome is common, as logistic models may inflate the risk estimates. Informed by prior literature and a prespecified causal diagram (Fig B in S1 Appendix), we adjusted the associations for child’s age, sex, and birth year, as well as maternal socio-demographic and clinical characteristics, including age at delivery, parity, country of birth, marital status, neighborhood income quintiles, preexisting psychiatric disorders, and pre-gestational diabetes. The analysis was carried out on males and females combined since no evidence of effect modification by sex was observed (p-for-interaction: 0.922). To identify subgroups of CHD pregnancies at a particularly high risk, we also tested potential effect modification by neighborhood income quintiles (as a proxy for maternal socioeconomic status) and pregnancy-induced disorders including ischemic placental disease and gestational diabetes. The effect modifications were evaluated on both multiplicative and additive scales, with additive interaction quantified using Relative Excess Risk due to Interaction (RERI) and the Attributable Proportion due to interaction [44].

#### Causal mediation analysis.

Maternal CHD was not associated with an increased risk of SGA in our preliminary investigation, and therefore, SGA was dropped from the final mediation analysis. Since maternal CHD is a risk factor for preterm birth which was also associated with developmental vulnerability (Tables 1 and D in S1 Appendix) [10,11,45], we used a counterfactual method to investigate the mediating role of preterm birth in the association between maternal CHD and child developmental vulnerability (Fig B in S1 Appendix) [46]. Given that preterm birth may also potentially interact with maternal CHD, we applied the four-way decomposition method using the Stata’s *med4way* command [47,48]. This allowed us to decompose the total excess relative risk into four components: controlled direct effect (due to neither mediation nor interaction), reference interaction (due to interaction only), mediated interaction (due to both mediation and interaction), and pure indirect effect (due to mediation only). The parameters were obtained by specifying a logistic model for the mediator and Poisson model for the outcome. The 95% CIs were calculated through bootstrapping, with 1,000 replications. The mediation analysis was based on the assumptions that there were no unmeasured confounders of the exposure-outcome, exposure-mediator, and mediator-outcome associations and no unmeasured mediator-outcome confounders influenced by the exposure [49].

**Table 1:** Maternal and child characteristics by maternal CHD status.

Maternal CHD			
	Total (*N* = 256,629)	No (*N* = 256,173)	Yes (*N* = 456)
**Child characteristics**	% (*n*)	% (*n*)	% (*n*)
Gestational age (years), mean ± SD	39 ± 1.7	39 ± 1.7	38 ± 1.9
Age (years), mean ± SD	5.6 ± 0.3	5.6 ± 0.3	5.6 ± 0.3
Sex			
Male	51.4 (131,833)	51.4 (131,602)	50.7 (231)
Female	48.6 (124,796)	48.6 (124,571)	49.3 (225)
Birth year			
1995–1999	13.4 (34,354)	13.4 (34,327)	5.9 (27)
2000–2004	28.1 (72,114)	28.1 (72,026)	19.3 (88)
2005–2009	26.4 (67,823)	26.4 (67,706)	25.7 (117)
2010–2014	26.2 (67,305)	26.2 (67,143)	35.5 (162)
2015–2016	5.9 (15,033)	5.8 (14,971)	13.6 (62)
Preterm birth (gestational weeks)			
No (≥37)	94.4 (242,202)	94.4 (241,792)	89.9 (410)
Yes (<37)	5.6 (14,427)	5.6 (14,381)	10.1 (46)
Birthweight-for-gestational age (percentile)			
SGA (<10th)	3.0 (7,735)	3.0 (7,724)	2.4 (11)
AGA (10th–90th)	72.4 (185,863)	72.4 (185,540)	70.8 (323)
LGA (≥90th)	24.6 (63,031)	24.6 (62,909)	26.8 (122)
Major malformations at birth			
No	97.8 (250,977)	97.8 (250,542)	95.4 (435)
Yes	2.2 (5,652)	2.2 (5,631)	4.6 (21)
Child CHD			
No	98.9 (253,764)	98.9 (253,318)	97.8 (446)
Yes	1.1 (2,865)	1.1 (2,855)	2.2 (10)
**Maternal characteristics**			
Age at delivery (years), mean ± SD	29.7 ± 5.5	29.7 ± 5.5	28.1 ± 6.0
Age at delivery (years)			
≤24	18.2 (46,769)	18.2 (46,634)	29.6 (135)
25–29	28.7 (73,688)	28.7 (73,567)	26.5 (121)
30–34	32.9 (84,524)	32.9 (84,392)	28.9 (132)
≥35	20.1 (51,648)	20.1 (51,580)	14.9 (68)
Parity			
1	45.6 (117,127)	45.6 (116,935)	42.1 (192)
2	37.3 (95,795)	37.3 (95,614)	39.7 (181)
3	12.3 (31,621)	12.3 (31,564)	12.5 (57)
≥4	4.7 (12,086)	4.7 (12 060)	5.7 (26)
Country of birth			
Canada	72.2 (185,357)	72.2 (184,961)	86.8 (396)
Europe	18.2 (46,788)	18.2 (46,751)	8.1 (37)
Asia	4.3 (11,078)	4.3 (11,068)	2.2 (10)
Others	5.2 (13,406)	5.2 (13,393)	2.9 (13)
Marital status			
Married	70.2 (180,071)	70.2 (179,825)	53.9 (246)
Never married	21.3 (54,609)	21.3 (54,454)	34.0 (155)
Single/Divorced/Widowed	3.7 (9,459)	3.7 (9,443)	3.5 (16)
Other	4.9 (12,490)	4.9 (12,451)	8.6 (39)
Neighborhood income quintiles			
1st (lowest)	21.1 (54,135)	21.1 (54,021)	25.0 (114)
2nd	21.5 (55,078)	21.5 (54,981)	21.3 (97)
3rd	20.8 (53,464)	20.8 (53,382)	18.0 (82)
4th	20.2 (51,910)	20.2 (51,821)	19.5 (89)
5th (highest)	16.4 (42,042)	16.4 (41,968)	16.2 (74)
BMI in early pregnancy (kg/m^2^)*			
Underweight (<18.5)	3.1 (7,949)	3.1 (7,933)	3.5 (16)
Normal weight (18.5–24.9)	35.4 (90,870)	35.4 (90,718)	33.3 (152)
Overweight (25–25.9)	12.9 (33,132)	12.9 (33,052)	17.5 (80)
Obese (≥30)	7.8 (20,108)	7.8 (20,067)	9.0 (41)
Missing	40.7 (104,570)	40.8 (104,403)	36.6 (167)
preexisting psychiatric disorders			
No	89.2 (228,894)	89.2 (228,518)	82.5 (376)
Yes	10.8 (27,735)	10.8 (27,655)	17.5 (80)
Pre-gestational diabetes			
No	97.1 (249,154)	97.1 (248,719)	95.4 (435)
Yes	2.9 (7,475)	2.9 (7,454)	4.6 (21)
Gestational diabetes			
No	92.7 (238,015)	92.7 (237,590)	93.2 (425)
Yes	7.3 (18,614)	7.3 (20,595)	6.8 (31)
Medication use in pregnancy^#^			
No	99.0 (254,036)	99.0 (253,594)	96.9 (442)
Yes	1.0 (2,593)	1.0 (2,579)	3.1 (14)
Ischemic placental disease^!^			
No	94.7 (243,077)	94.7 (242,653)	93.0 (424)
Yes	5.3 (13,552)	5.3 (13,520)	7.0 (32)
Mode of delivery			
Cesarean delivery	28.3 (72,685)	28.3 (72,536)	32.7 (149)
Spontaneous vaginal delivery	5.6 (14,488)	5.6 (14,465)	5.0 (23)
Instrumental vaginal delivery	66.0 (169,266)	66.0 (168,982)	62.3 (284)
Missing	0.1 (190)	0.1 (190)	0.0 (0)

Note: AGA, Appropriate-for-gestational age; BMI, Body Mass Index; CHD, Congenital heart disease; LGA, Large-for-gestational age; SD, Standard deviation; SGA, Small-for-gestational age.

*Data on maternal BMI were obtained from the BC Perinatal Data Registry available from 2000 (*N* = 222,275).

#Medications include beta blockers and calcium channel blockers.

!Ischemic placental disease is a composite of three variables: placental abruption, preeclampsia, and delivery of an SGA infant.

#### Sensitivity analyses.

A number of sensitivity analyses were conducted. First, given that information on maternal BMI was available through the BC Perinatal Data Registry since 2000 and had a high proportion of missing data (31.6%), multiple imputation analysis was performed to evaluate the strength of confounding due to maternal pre-pregnancy BMI. Under the assumption of missing at random, we generated 35 imputed datasets (proportional to the total missing of 35%) using the multiple imputation with chained equations approach [50] and reported the pooled estimates. Second, we excluded children diagnosed with CHD or any major congenital malformations to partly evaluate whether the observed associations were robust to potential genetic confounding. Third, considering the heterogeneity of maternal CHD, additional analysis was conducted to examine the association between severity of maternal CHD (mild, moderate, and severe) and child developmental vulnerability. Fourth, we presented the association separately for the ICD-9 and ICD-10 codes to explore whether the differences in the two coding systems have any implications for the key findings. Fifth, we further adjusted for maternal use of cardiac medications during pregnancy [51] in a sensitivity analysis. Sixth, we repeated the analysis after excluding the EDI records collected prior to 2004 to account for the change in response format introduced from wave 2 onward. Seventh, since immigrant women were less likely to receive a CHD diagnosis than those born in Canada, we conducted a sensitivity analysis restricted to children of Canadian-born mothers. Eighth, because outpatient CHD diagnoses not supported cardiology-specific claims were excluded from our primary CHD definition, we additionally excluded such cases from the unexposed group to improve diagnosis specificity. Ninth, to minimize exposure misclassification, we further conducted a sensitivity analysis excluding ICD-10 code Q21.1 and ICD-9 code 745.5 from the maternal CHD definition, as these codes may capture patent foramen ovale (PFO), a common anatomical variant rather than true CHD. Tenth, since children who died before EDI assessment were likely more vulnerable, we treated death before age 5 years (derived from BC Vital Events and Statistics – Deaths) as developmental vulnerability to assess potential survivor bias. The three sensitivity analyses described above (i.e., 8th to 10th) were added during peer review in response to reviewer comments.

#### Protocol and analysis plan.

The study did not have a prospectively registered protocol or analysis plan. The inclusion/exclusion criteria and initial analyses were planned a priori based on study aims. The mediation analysis was partly data-informed, with mediators selected based on their observed associations with both the exposure and the outcome. Additional sensitivity analyses conducted during the peer review process did not change the main conclusions. The study is reported as per the RECORD (Reporting of Studies Conducted Using Observational Routinely-Collected Data) guideline (S1 Checklist).

#### Ethical statement.

The study received ethical approval from the Ethics Committee at the University of British Columbia, Canada, with a waiver of informed consent for the administrative data (approval number: H20-00902). For the EDI survey data, informed passive consent was obtained prior to data collection from the parents/guardians who could choose to opt their children out of participation.

## Results

A total of 256,629 kindergarten school children (female 48.6%) with available survey data on early childhood development were included in the final analysis. Children in the study were born at the mean gestational age of 39 weeks and were on average 5.6 years old at the time of EDI data collection. Among all children, 456 (0.2%) were exposed to maternal CHD, with prevalence increasing over the study period (Fig A in S1 Appendix).

Children exposed to maternal CHD had higher prevalence of preterm birth and major congenital malformations compared with the unexposed (Table 1). About 2.2% children born to women with a history of CHD were themselves diagnosed with a CHD, compared with 1.1% in children of mothers without CHD. Compared with women without CHD, those with CHD were younger at delivery and were more frequently Canadian-born, unmarried, and from lower income neighborhoods. They also had higher prevalence of overweight status, preexisting psychiatric disorders, pre-gestational diabetes, ischemic placental disease, cardiac medication use during pregnancy, and cesarean delivery.

The proportion of children experiencing developmental vulnerability in two or more domains was 25.2% among women with CHD compared with 16.6% among women without CHD (Table 2). Children exposed to maternal CHD consistently showed a higher proportion of developmental vulnerability across all domains of child development. In the adjusted analysis, the exposed children had a 28% higher risk of developmental vulnerability in at least two domains (aRR 1.28; 95% CI [1.11, 1.48]) compared with the unexposed. Of the five domains of development, an increased risk of vulnerability was observed for physical health and wellbeing, social competence, language and cognitive development, and communication skill and general knowledge (Table 2).

**Table 2:** Prevalence and risk ratios of child developmental vulnerability by maternal CHD status.

Maternal CHD					
**Child developmental outcomes**	**Total (*N* = 256,629)**	**No (*N* = 256,173)**	**Yes (*N* = 456)**	**Crude**	Adjusted*
	% (*n*)	% (*n*)	% (*n*)	RR (95% CI)	RR (95% CI)
**Overall developmental vulnerability** ^#^					
>10th percentile	83.3 (213,893)	83.4 (213,552)	74.8 (341)	1.00 (Ref.)	1.00 (Ref.)
≤10th percentile	16.7 (42,736)	16.6 (42,621)	25.2 (115)	1.52 (1.29, 1.78)	1.28 (1.11, 1.48)
**Physical health and wellbeing**					
>10th percentile	86.6 (222,184)	86.6 (221,828)	78.1 (356)	1.00 (Ref.)	1.00 (Ref.)
≤10th percentile	13.4 (34,445)	13.4 (34,345)	21.9 (100)	1.64 (1.38, 1.95)	1.31 (1.11, 1.54)
**Emotional maturity**					
>10th percentile	86.0 (220,732)	86.0 (220,365)	80.5 (367)	1.00 (Ref.)	1.00 (Ref.)
≤10th percentile	14.0 (35,897)	14.0 (35,808)	19.5 (89)	1.40 (1.16, 1.68)	1.14 (0.96, 1.37)
**Social competence**					
>10th percentile	86.1 (220,954)	86.1 (220,589)	80.0 (365)	1.00 (Ref.)	1.00 (Ref.)
≤10th percentile	13.9 (35,675)	13.9 (35,584)	20.0 (91)	1.44 (1.20, 1.73)	1.22 (1.02, 1.45)
**Language and cognitive development**					
>10th percentile	90.3 (231,726)	90.3 (231,342)	84.2 (384)	1.00 (Ref.)	1.00 (Ref.)
≤10th percentile	9.7 (24,903)	9.7 (24,831)	15.8 (72)	1.63 (1.32, 2.01)	1.39 (1.13, 1.70)
**Communication and general knowledge**					
>10^th^ percentile	87.7 (225,157)	87.7 (224,775)	83.8 (382)	1.00 (Ref.)	1.00 (Ref.)
≤10th percentile	12.3 (31,472)	12.3 (31,398)	16.2 (74)	1.32 (1.07, 1.63)	1.33 (1.09, 1.63)

Note: CHD, Congenital heart disease; CI, Confidence interval; RR, Risk ratio.

#Defined as bottom 10th percentile score on two or more domains of development, based on a BC provincial cutoff from a normed sample.

All models compared the outcome between children with and without maternal CHD (Reference = 1.00).

*Adjusted for child’s sex, birth year, and age at EDI completion, and maternal age at delivery, parity, country of birth, marital status, neighborhood income quintiles, preexisting psychiatric disorders, and pre-gestational diabetes.

**Fig 2:**
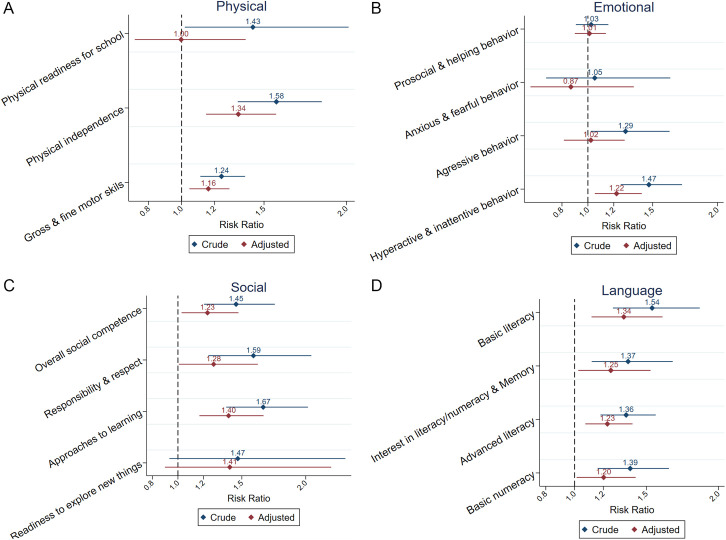
Risk ratios for the associations between maternal CHD and subdomains of child developmental vulnerability. Note: CHD, Congenital heart disease. The sample size for this analysis slightly varies across subdomains due to the exclusion of outcome-specific missing data (see also Table E in S1 Appendix). The adjusted model included child’s sex, birth year, and age at EDI completion, and maternal age at delivery, parity, country of birth, marital status, neighborhood income quintiles, preexisting psychiatric disorders, and pre-gestational diabetes.

Table 3 shows the associations between maternal CHD and the severity of child developmental vulnerability. The relative risks were generally elevated with the increasing number of vulnerable domains, ranging from aRR 1.16 for one or more domains (95% CI [1.04, 1.29]) to aRR 1.64 (95% CI [1.09, 2.45]) when all five domains were affected. Analysis of EDI subdomains further showed that maternal CHD was associated with increased risk of vulnerability across multiple areas, including physical independence, gross and fine motor skills, hyperactive and inattentive behavior, overall social competence, responsibility and respect, approaches to learning, basic literacy, interest in literacy or numeracy and memory, advanced literacy, and basic numeracy (Fig 2 and Table E in S1 Appendix).

**Table 3:** Risk ratios for the associations between maternal CHD and severity of child developmental vulnerability.

		Children with vulnerability % (*n*)	Crude	Adjusted*
RR (95% CI)	RR (95% CI)
**≥1 Domain**	**Maternal CHD**			
	No	29.7 (76,001)	1.00 (Ref.)	1.00 (Ref.)
	Yes	38.4 (175)	1.29 (1.15, 1.45)	1.16 (1.04, 1.29)
**≥2 Domains**	**Maternal CHD**			
	No	16.6 (42,621)	1.00 (Ref.)	1.00 (Ref.)
	Yes	25.2 (115)	1.52 (1.29, 1.78)	1.28 (1.11, 1.48)
**≥3 Domains**	**Maternal CHD**			
	No	9.5 (24,353)	1.00 (Ref.)	1.00 (Ref.)
	Yes	15.1 (69)	1.59 (1.28, 1.98)	1.30 (1.06, 1.60)
**≥4 Domains**	**Maternal CHD**			
	No	5.1 (13,157)	1.00 (Ref.)	1.00 (Ref.)
	Yes	9.9 (45)	1.92 (1.46, 2.56)	1.54 (1.18, 2.00)
**5 Domains**	**Maternal CHD**			
	No	2.3 (5,834)	1.00 (Ref.)	1.00 (Ref.)
	Yes	4.8 (22)	2.12 (1.41, 3.19)	1.64 (1.09, 2.45)

Note: CHD, Congenital heart disease; CI, Confidence interval; RR, Risk ratio.

*Adjusted for child’s sex, birth year, and age at EDI completion, and maternal age at delivery, parity, country of birth, marital status, neighborhood income quintiles, preexisting psychiatric disorders, and pre-gestational diabetes.

**Fig 3:**
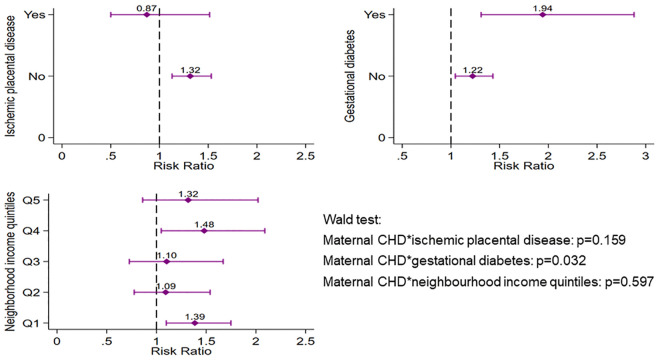
Effect modification by maternal pregnancy complications and neighborhood income quintiles on the association between maternal CHD and child developmental vulnerability, multiplicative scale. Note: CHD, Congenital heart disease. The Risk Ratios were adjusted for child’s sex, birth year, and age at EDI completion, and maternal age at delivery, parity, country of birth, marital status, neighborhood income quintiles, preexisting psychiatric disorders, and pre-gestational diabetes.

The four-way decomposition analysis (Table F in S1 Appendix) showed that the pure indirect effect via preterm birth accounted for about 8.0% of the total excess relative risk, with no evidence of interaction between maternal CHD and gestational age. The association was not modified by maternal ischemic placental disease and neighborhood income quintiles (Fig 3). However, the association was stronger in children exposed to both maternal CHD and gestational diabetes (aRR 1.94; 95% CI [1.31, 2.88]; Fig 3) compared with those exposed to maternal CHD alone (aRR 1.22; 95% CI [1.04, 1.43]), with a statistically significant difference (interaction *p*-value: 0.032). On the additive scale, the interaction did not reach statistical significance (Table G in S1 Appendix), with an estimated RERI of 0.90 (95% CI [−0.03, 1.82]).

### Sensitivity analyses.

The association between maternal CHD and child developmental vulnerability in the multiple imputation analysis was very similar to the primary analysis, even after adjustment for maternal BMI (Table H in S1 Appendix). Exclusion of children with CHD and other major congenital malformations attenuated the association by 15% (Table I in S1 Appendix). Maternal severe CHD was more strongly associated with child developmental vulnerability (aRR 1.98; 95% CI [1.31, 3.00]; Table J in S1 Appendix) in comparison to mild CHD (aRR 1.19; 95% CI [1.00, 1.42]), with a statistically significant difference between the two associations (ratio of aRRs: 1.66; 95% CI [1.06, 2.61]). The magnitude of the association remained consistent regardless of whether maternal CHD was defined using ICD-9 or ICD-10 codes (Table K in S1 Appendix). Adjustment for cardiac medications use in pregnancy led to minimal attenuation of the association (Table L in S1 Appendix). The RRs were elevated in analyses restricted to children of Canadian-born mothers (Table M in S1 Appendix) and to those participating in EDI survey from wave 2 onward (Table N in S1 Appendix). Excluding outpatient CHD diagnoses without cardiology-specific claims (*n* = 1,590) and an alternative CHD definition excluding potential PFO cases (*n* = 16) yielded similar effect estimates (Tables O and P in S1 Appendix). Finally, in sensitivity analysis assuming death before age 5 as developmental vulnerability, the association was similar in magnitude to the primary analysis (Table Q in S1 Appendix).

## Discussion

This population-based investigation shows that exposure to maternal CHD was associated with an increased risk of developmental vulnerability at early school age. The association was consistently observed across a wide range of developmental domains and subdomains, including physical, socio-emotional, and cognitive outcomes. Preterm birth mediated only a small proportion of the association. Children exposed to both maternal CHD and comorbid gestational diabetes had a higher risk of vulnerability compared with those exposed to either condition alone. In addition, maternal severe CHD as compared to mild CHD was more strongly associated with child developmental vulnerability.

Direct comparisons of our study findings are limited as evidence is scarce on the impact of maternal CHD specifically on childhood developmental vulnerability. However, the results are consistent with our earlier work, based on Swedish and Canadian populations, which demonstrated that different forms of preexisting maternal heart disease including CHD were associated with an increased risk of offspring’s attention-deficit hyperactive disorder and autism spectrum disorder [52]. Similarly, other studies have shown poor developmental outcomes in children exposed to various maternal disorders such as obesity [53], diabetes [54], and preeclampsia [55], implying the broader relevance of maternal health for prenatal origin of child’s suboptimal physical and mental development. Our finding that mild maternal CHD was associated with smaller increase in risk than severe CHD aligns with previous research reporting lower risks of obstetric and neonatal complications among women with mild versus moderate or severe CHD [56].

The mechanisms underlying the observed association between maternal CHD and early developmental vulnerability are likely multifactorial. One potential pathway involves impairment of placental structure and function [17,57,58]. Pregnancy requires substantial increases in maternal cardiac output to support uteroplacental circulation and facilitate maternal-fetal exchange of nutrients and oxygen [17]. In women with CHD, compromised cardiac function may decrease cardiac output and uteroplacental perfusion [17,57], potentially altering the fetal growth and development trajectory [59].

In line with this, prior epidemiological studies have linked maternal CHD to placental dysfunction and adverse obstetric and neonatal complications [16–18,60]. However, in the present study, we found limited evidence that maternal ischemic placental disease and preterm delivery mediated or modified the risk of offspring developmental vulnerability in mothers with CHD. In contrast, the risk was amplified when maternal CHD co-occurred with gestational diabetes, suggesting a possible interaction through the intrauterine pathways. Gestational diabetes is characterized by maternal hyperglycemia, which may lead to fetal hyperinsulinemia, oxidative stress, and chronic inflammation [61–63]. These metabolic disturbances may impose additional strain on the placenta and fetus in CHD pregnancies, exacerbating placental dysfunction and disruptions in fetal growth and brain development [54,63,64]. Although biologically plausible, this potential interaction requires confirmation in larger studies.

We also observed a higher prevalence of CHD among children of mothers with CHD, consistent with familial aggregation and inherited susceptibility [65]. Certain genetic disorders, such as Down syndrome (trisomy 21), Noonan syndrome, and 22q11.2 deletion [66], are known to be associated with both CHD and neurodevelopmental delays [58,67], and therefore may contribute to the observed association. While the genetic syndromes could not be accounted for in our analysis, excluding children with CHD diagnoses and other congenital malformations had minimal impact on our results. This suggests that overt cardiac anomalies are unlikely to fully account for the findings, although it remains possible that unmeasured genetic factors may contribute to child developmental outcomes even in the absence of structural heart defects [68]. Notably, previous studies have found no association between paternal CHD and adverse birth outcomes, despite reduced fertility rate among men with CHD [69,70]. In contrast, the associations for maternal CHD supports the hypothesis that intrauterine conditions, rather than genetic transmission alone, may cause adverse outcomes in offspring.

Finally, the postnatal social pathway may also play a role. Adults with CHD may experience lower educational attainment, reduced income, lower rate of marriage or long-term partnerships, poorer neuropsychological functioning, and diminished health-related quality of life [67]. These disadvantages can shape the quality of the postnatal family environment, thereby affecting child development. Taken together, our findings likely reflect a combination of intrauterine, genetic, and social pathways [67].

A key strength of the study is the integration of the large, population-based, and well-validated EDI surveys with multiple administrative data sources including birth records, hospital and outpatient records, medication dispensations, and census information. The comprehensive linkage provided detailed information on a wide range of maternal socio-demographic and clinical factors, thereby reducing the risk of residual confounding. In addition, the relatively large sample and holistic assessment of school readiness enabled analysis of 16 distinct subdomains of child development separately, providing a nuanced understanding of how maternal CHD may affect specific development aspects.

Some limitations should also be acknowledged. First, the prevalence of maternal CHD in pregnancy (0.2%) in our study was lower than the estimated prevalence among live births (1.1%). This may reflect lower likelihood of childbearing among women with moderate or severe CHD [6], lower CHD survival among women born in earlier calendar periods, and underdiagnosis of mild or asymptomatic CHD, particularly if diagnosed in childhood and not requiring ongoing care. Although our estimate is broadly comparable with previous literature reporting 0.07% to 0.3% of CHD in pregnancy cohorts [5–7,10,71], some degree of exposure misclassification is possible (e.g., CHD diagnoses outside of BC) and would likely bias the observed association toward the null. Similarly, our measure of CHD complexity is crude as it was derived from ICD-codes and may not fully capture clinically meaningful distinctions. More detailed clinical classification is needed in future research to clarify which phenotypes of CHD more strongly influence child development and to explore the underlying mechanisms. Second, in the absence of data on genetic factors and postnatal family environment, we could not fully distinguish intrauterine effects from shared genetic and familial influences. Third, the EDI data were not available for all kindergarten children due to the multi-year wave-based design of the survey, with approximately one-third of the schools participating each year. This is unlikely to have introduced bias since school selection was unrelated to maternal CHD status. However, if maternal CHD affects fetal and early childhood mortality, the true association might be underestimated due to restricting the study population to children who survived to kindergarten [72], although we partly addressed this concern in a sensitivity analysis by treating death before age 5 as developmental vulnerability. Fourth, although the overall sample size was large, insufficient statistical power within certain strata of potential effect modifiers restricted our ability to detect interaction. Fifth, the EDI data were primarily collected in public schools across BC while the home-schooled children and those who attended private schools were rarely captured, which may limit generalizability.

In conclusion, this population-based cohort study from BC, Canada, suggests that children exposed to maternal CHD were at increased risk of being developmentally vulnerable at school entry across multiple domains. As the population of pregnant women with preexistent CHD is expanding globally, these findings highlight the importance of recognizing maternal CHD as a potential risk factor for child developmental disadvantage and the need of a life course approach to understanding its consequences beyond pregnancy. While children of mothers with CHD may benefit from early developmental monitoring, optimizing preconception and perinatal care for affected women may help reduce the risk of adverse long-term health and developmental outcomes in offspring.

## Supporting information

S1 Appendix Supplementary figures and tables. **Fig A.** Prevalence of maternal CHD by child birth year, 1995−2016. **Fig B.** Illustration of the causal mediation framework of the study. **Table A.** Classification of maternal adult CHD with International Classification of Disease (ICD) codes. **Table B**. Wave-specific participation rate in the Early Development Instrument Survey in British Columbia, Canada, 1999−2022. **Table C.** International Classification of Disease (ICD) codes used to define maternal and child clinical covariates. **Table D.** Risk ratios for the associations of potential mediators or effect modifiers with child developmental vulnerability, British Columbia, Canada,1995−2016. **Table E.** Distribution of the subdomains of child developmental vulnerability by maternal adult CHD status. **Table F.** Four-way decomposition of the association between maternal CHD and child developmental vulnerability with preterm birth as mediator and effect modifier. **Table G.** Effect modification by maternal pregnancy complications and neighborhood income quintiles on the association between maternal CHD and early childhood developmental vulnerability, additive scale. **Table H.** Risk ratios from multiple imputation analysis for the association between maternal CHD and child’s overall developmental vulnerability, with additional adjustment for maternal BMI. **Table I.** Sensitivity analysis of the association between maternal CHD and child’s overall developmental vulnerability, after excluding child CHD and major congenital malformations. **Table J.** Risk ratios for the association between maternal CHD severity and child’s overall developmental vulnerability. **Table K.** Risk ratios for the associations between maternal CHD and child’s overall developmental vulnerability, stratified by ICD-9 versus ICD-10 coding systems. **Table L.** Risk ratios for the association between maternal CHD and child’s overall developmental vulnerability, with additional adjustment for maternal use of cardiac medications in pregnancy. **Table M.** Sensitivity analysis of the association between maternal CHD and child’s overall developmental vulnerability, after excluding children of foreign-born mothers. **Table N.** Sensitivity analysis of the association between maternal CHD and child’s overall developmental vulnerability, after excluding children participating in the pilot or Wave 1 survey. **Table O.** Sensitivity analysis of the association between maternal CHD and child’s overall developmental vulnerability, excluding outpatient CHD diagnoses without a cardiology-specific claims. **Table P.** Sensitivity analysis of the association between maternal CHD and child’s overall developmental vulnerability, excluding potential Patent Foramen Ovale cases. **Table Q.** Sensitivity analysis of the association between maternal CHD and child’s overall developmental vulnerability, with death before age-5 treated as developmental vulnerability.
(DOCX)

S1 Code Stata code for analysis. (DO)

S1 Checklist REporting of studies Conducted using Observational Routinely-collected Data (RECORD) checklist. (DOCX)

## Abbreviations

ATC: anatomical therapeutic chemical
BC: British Columbia
CHD: congenital heart disease
CI: confidence intervals
DAD: discharge abstract database
EDI: early development instrument
ESC: European Society of Cardiology
ICD: International Classification of Disease
MSP: medical services plan
PCCF: postal code conversion file
PFO: patent foramen ovale
RECORD: Reporting of Studies Conducted Using Observational Routinely-Collected Data
RERI: relative excess risk due to interaction
RR: risk ratios
SGA: small-for-gestational age.
