# Supplementary material for: Maternal congenital heart disease and risk of child developmental vulnerability in early school age: A population-based cohort study
Source: PLoS Med. 2026 Jul 2;23(7):e1004890. doi: 10.1371/journal.pmed.1004890 (PMC13327283; doi:10.1371/journal.pmed.1004890)
Supplement: S1 Appendix — Fig A. Prevalence of maternal CHD by child birth year, 1995−2016. Fig B. Illustration of the causal mediation framework of the study. Table A. Classification of maternal adult CHD with International Classification of Disease (ICD) codes. Table B. Wave-specific participation rate in the Early Development Instrument Survey in British Columbia, Canada, 1999−2022. Table C. International Classification of Disease (ICD) codes used to define maternal and child clinical covariates. Table D. Risk ratios for the associations of potential mediators or effect modifiers with child developmental vulnerability, British Columbia, Canada,1995−2016. Table E. Distribution of the subdomains of child developmental vulnerability by maternal adult CHD status. Table F. Four-way decomposition of the association between maternal CHD and child developmental vulnerability with preterm birth as mediator and effect modifier. Table G. Effect modification by maternal pregnancy complications and neighborhood income quintiles on the association between maternal CHD and early childhood developmental vulnerability, additive scale. Table H. Risk ratios from multiple imputation analysis for the association between maternal CHD and child’s overall developmental vulnerability, with additional adjustment for maternal BMI. Table I. Sensitivity analysis of the association between maternal CHD and child’s overall developmental vulnerability, after excluding child CHD and major congenital malformations. Table J. Risk ratios for the association between maternal CHD severity and child’s overall developmental vulnerability. Table K. Risk ratios for the associations between maternal CHD and child’s overall developmental vulnerability, stratified by ICD-9 versus ICD-10 coding systems. Table L. Risk ratios for the association between maternal CHD and child’s overall developmental vulnerability, with additional adjustment for maternal use of cardiac medications in pregnancy. Table M. Sensitivity analysis of the assoc [file pmed.1004890.s001.docx]

**S1 Appendix: Supplementary figures and tables**

Hossin et al. Maternal congenital heart disease and risk of child developmental vulnerability in early school age: A population-based cohort study


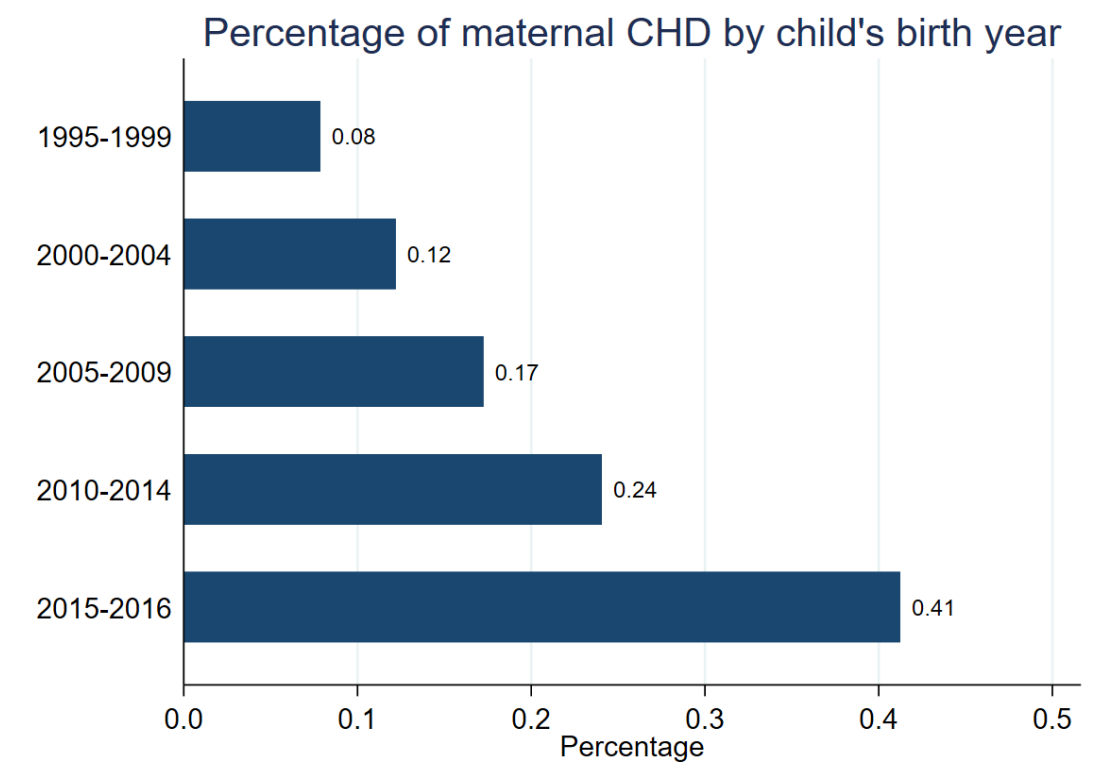


**Fig A.** Prevalence of maternal CHD by child birth year, 1995-2016.

Note: CHD, Congenital heart disease.


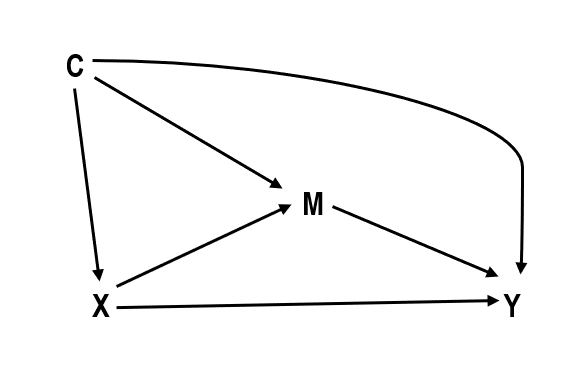


**Fig B.** Illustration of the causal mediation framework of the study

X = Exposure: maternal CHD status in pregnancy; Y = Outcome: child developmental vulnerability in kindergarten; M = Mediator: preterm birth; C = Confounders: child’s sex, birth year, and age at EDI completion, as well as maternal age at delivery, parity, country of birth, marital status, neighborhood income quintiles, pre-existing psychiatric disorders, and pre-gestational diabetes. Maternal country of birth was treated as a cofounder affecting exposure-mediator, exposure-outcome, and mediator-outcome associations, while the remaining set of confounders were treated as mediator-outcome confounders.

| **Table A.** Classification of maternal adult CHD with International Classification of Disease (ICD) codes. | | |
| --- | --- | --- |
| **Maternal CHD** | **ICD-10** | **ICD-9** |
| Overall CHD | Q20-Q26 | 745, 746, 747.1, 747.2, 747.3, 747.4 |
| Mild | Q21.0, Q21.1,  Q22.1, Q22.2, Q22.3, Q22.8,  Q23.0, Q23.1, Q23.2, Q23.3, Q23.8, Q23.9,  Q25.0, Q25.6,  Q26.1 | 745.4, 745.5,  746.02, 746.09, 746.00, 746.09,  747.0, 747.39, 747.49 |
| Moderate | Q20.3,  Q21.2, Q21.3, Q21.4, Q21.8,  Q22.1, Q22.4, Q22.5,  Q23.3,  Q24.2, Q24.3, Q24.4, Q24.5, Q24.8, Q24.9,  Q25.1, Q25.3, Q25.4, Q25.7,  Q26.2, Q26.3, Q26.4,  Q87.4,  Q96.9 | 745.0, 745.2, 745.6,  746.1, 746.2, 746.3, 746.4, 746.81, 746.85, 746.86, 746.89, 746.9  747.10, 747.22, 747.41, 747.42 |
| Severe | Q20.0, Q20.1, Q20.2, Q20.3, Q20.4, Q20.5, Q20.6, Q20.8, Q20.9,  Q22.0, Q22.6,  Q23.4,  Q24.6, Q24.8, Q24.0,  Q25.2, Q25.5,  Q89.3 | 745.10, 745.11, 745.12, 745.19, 745.3, 745.2,  745.8,  746.01, 746.7,  747.11, 747.29, 747.31,  756.89,  759.0 |

Note: CHD, Congenital heart disease; ICD, International Classification of Disease.

| **Table B**. Wave-specific participation rate in the Early Development Instrument (EDI) survey in British Columbia, Canada, 1999-2022. | | | |
| --- | --- | --- | --- |
| Wave | EDI number | Enrolment number | Participation Rate |
| Wave 1 | 40,772 | - | - |
| Wave 2 | 37,229 | 37,813 | 98.5 |
| Wave 3 | 36,995 | 37,724 | 98.1 |
| Wave 4 | 46,204 | 47,660 | 96.9 |
| Wave 5 | 41,337 | 44,873 | 92.1 |
| Wave 6 | 41,453 | 48,072 | 86.2 |
| Wave 7 | 42,184 | 47,130 | 89.5 |
| Wave 8 | 40,242 | 45,604 | 88.2 |
| Note: Enrolment data is provided by the Ministry of Education and Child Care in British Columbia. Information on the number of school enrolment during Wave 1 survey is not available. | | | |

| **Table C.** International Classification of Disease (ICD) codes used to define maternal and child clinical covariates. | | | |
| --- | --- | --- | --- |
| **Maternal covariates** | **ICD-10** | **ICD-9** | **Data source** |
| Pre-existing psychiatric disorders | F00-F99 | 290-319; 50B | DAD + MSP |
| Pre-gestational diabetes | E10-E14, O240, O241, O243 | 250, 6480 | DAD + MSP |
| Gestational diabetes | O244 | 6488 | DAD + MSP + PDR |
| Placental abruption | O450, O458, O459 | 6412 | DAD + MSP + PDR |
| Preeclampsia | O14, O15 | 6424, 6425, 6426 | DAD + MSP + PDR |
| Medication use during pregnancy (ATC codes) * |  |  |  |
| Betablockers | C07AB02, C07AB07, C07AB03, C07AG02, C07AG01 | | PharmaNet |
| Calcium channel blockers | C08DA01, C08CA01, C08CA05, C08CA02, C08DB01, C08CA06 | | PharmaNet |
| **Child covariates** |  |  |  |
| Major congenital malformations | Q7550, Q175, Q180, Q181, Q249, Q250, Q270, Q289, Q314, Q315, Q320, Q381, Q523, Q53, Q65, Q665, Q666, Q667, Q668, Q669, Q690, Q699, Q703, Q760, Q799, Q825, Q829 | 744B, 744E, 746X, 747F, 750A, 750B, 752F, 754D, 754G, 755G, 755A, 755B, 756B, 756X, 757D, 757X. | DAD + MSP |
| Congenital heart disease | Q20-Q26 | 745-747 | DAD + MSP |
| Note: ATC, Anatomical therapeutic chemical; DAD, Discharge abstract database; ICD, International Classification of Disease; MSP, Medical services plan; PDR, Perinatal data registry.  *Information on maternal medication use was obtained through dispensed drugs using the ATC codes available since 1996 in BC, Canada. | | | |

| **Table D.** Risk ratios for the associations of potential effect modifiers or mediators with child developmental vulnerability. | | | | |
| --- | --- | --- | --- | --- |
| **Potential effect modifiers/ mediators** | **Outcome: Child developmental vulnerability** | | |  |
|  | **Children with vulnerability**  % (n) | **Crude** | **Adjusted*** |  |
|  |  | RR (95% CI) | RR (95% CI) |  |
| Neighbourhood income quintiles |  |  |  |  |
| Q1 | 22.5 (12,186) | 1.82 (1.76, 1.87) | 1.47 (1.43, 1.51) |  |
| Q2 | 18.0 (9,935) | 1.46 (1.41, 1.50) | 1.28 (1.24, 1.32) |  |
| Q3 | 15.3 (8,199) | 1.24 (1.20, 1.28) | 1.17 (1.13, 1.20) |  |
| Q4 | 13.9 (7,205) | 1.12 (1.08, 1.16) | 1.10 (1.06, 1.13) |  |
| Q5 | 12.4 (5,211) | 1.00 | 1.00 |  |
| Ischemic placental disease |  |  |  |  |
| No | 16.2 (39,726) | 1.00 | 1.00 |  |
| Yes | 22.2 (3,010) | 1.36 (1.32, 1.40) | 1.32 (1.27, 1.36) |  |
| Gestational diabetes |  |  |  |  |
| No | 16.4 (38,677) | 1.00 | 1.00 |  |
| Yes | 19.7 (4,059) | 1.20 (1.17, 1.24) | 1.19 (1.15, 1.23) |  |
| Preterm birth |  |  |  |  |
| No | 16.2 (39,305) | 1.00 | 1.00 |  |
| Yes | 23.8 (3,431) | 1.47 (1.42, 1.51) | 1.31 (1.27, 1.35) |  |
| Note: CI, Confidence interval; CHD, Congenital heart disease; Q, Quintile; RR, Risk ratio.  *Adjusted for child’s sex, birth year, and age at EDI completion, as well as maternal CHD, age at delivery, parity, country of birth, marital status, neighbourhood income quintiles, pre-existing psychiatric disorders, and pre-gestational diabetes. | | | |  |

| **Table E.** Distribution of the subdomains of child developmental vulnerability by maternal CHD status. | | | | |
| --- | --- | --- | --- | --- |
| **Domains** | **Subdomains of child developmental vulnerability** | **Total** (N=256,629) | **Maternal CHD** | |
|  |  |  | No (N=256,173) | Yes (N=456) |
|  |  | % (n) | % (n) | % (n) |
| **Physical health and wellbeing** | Physical readiness for school |  |  |  |
|  | Ready for school | 95.2 (244,279) | 95.2 (243,854) | 93.2 (425) |
|  | Not ready for school | 4.7 (12,180) | 4.7 (12,149) | 6.8 (31) |
|  | Missing | 0.1 (170) | 0.1 (170) | 0.0 (0) |
|  | Physical independence |  |  |  |
|  | Ready for school | 84.3 (216,377) | 84.3 (216,033) | 75.4 (344) |
|  | Not ready for school | 15.6 (40,023) | 15.6 (39,911) | 24.6 (112) |
|  | Missing | 0.1 (229) | 0.1 (229) | 0.0 (0) |
|  | Gross & Fine motor skills |  |  |  |
|  | Ready for school | 66.6 (170,857) | 66.6 (170,590) | 58.6 (267) |
|  | Not ready for school | 33.4 (85,621) | 33.3 (85,432) | 41.4 (189) |
|  | Missing | 0.1 (151) | 0.1 (151) | 0.0 (0) |
| **Emotional maturity** | Prosocial & helping behaviour |  |  |  |
|  | Ready for school | 60.0 (153,875) | 60.0 (153,604) | 59.4 (271) |
|  | Not ready for school | 34.5 (88,457) | 34.5 (88,295) | 35.5 (162) |
|  | Missing | 5.6 (14,297) | 5.6 (14,274) | 5.0 (23) |
|  | Anxious & fearful behaviour |  |  |  |
|  | Ready for school | 96.0 (246,242) | 96.0 (245,806) | 95.6 (436) |
|  | Not ready for school | 4.0 (10,170) | 4.0 (10,151) | 4.2 (19) |
|  | Missing | 0.1 (217) | 0.1 (216) | <0.5 (<5) |
|  | Aggressive behaviour |  |  |  |
|  | Ready for school | 89.6 (229,877) | 89.6 (229,482) | 86.6 (395) |
|  | Not ready for school | 10.4 (26,596) | 10.4 (26,535) | 13.4 (61) |
|  | Missing | 0.1 (156) | 0.1 (156) | 0.0 (0) |
|  | Hyperactive & inattentive behaviour |  |  |  |
|  | Ready for school | 83.1 (213,340) | 83.1 (212,997) | 75.2 (343) |
|  | Not ready for school | 16.9 (43,265) | 16.8 (43,152) | 24.8 (113) |
|  | Missing | 0.0 (24) | 0.0 (24) | 0.0 (0) |
| **Social competence** | Overall social competence |  |  |  |
|  | Ready for school | 86.8 (222,768) | 86.8 (222,399) | 80.9 (369) |
|  | Not ready for school | 13.1 (33,657) | 13.1 (33,570) | 19.1 (87) |
|  | Missing | 0.1 (204) | 0.1 (204) | 0.0 (0) |
|  | Responsibility & respect |  |  |  |
|  | Ready for school | 92.5 (237,379) | 92.5 (236,978) | 87.9 (401) |
|  | Not ready for school | 7.5 (19,161) | 7.5 (19,107) | 11.8 (54) |
|  | Missing | 0.0 (89) | 0.0 (88) | <0.2 (<5) |
|  | Approaches to learning |  |  |  |
|  | Ready for school | 88.7 (227,526) | 88.7 (227,156) | 81.1 (370) |
|  | Not ready for school | 11.3 (29,066) | 11.3 (28,980) | 18.9 (86) |
|  | Missing | 0.0 (37) | 0.0 (37) | 0.0 (0) |
|  | Readiness to explore new things |  |  |  |
|  | Ready for school | 97.0 (248,940) | 97.0 (248,503) | 95.8 (437) |
|  | Not ready for school | 2.7 (6,900) | 2.7 (6,882) | 3.9 (18) |
|  | Missing | 0.3 (789) | 0.3 (788) | <0.5 (<5) |
| **Language and cognitive development** | Basic literacy |  |  |  |
|  | Ready for school | 88.0 (225,919) | 88.0 (225,546) | 81.8 (373) |
|  | Not ready for school | 11.8 (30,336) | 11.8 (30,253) | 18.2 (83) |
|  | Missing | 0.1 (374) | 0.1 (374) | 0.0 (0) |
|  | Interest in literacy or numeracy and Memory |  |  |  |
|  | Ready for school | 86.8 (222,852) | 86.8 (222,475) | 82.7 (377) |
|  | Not ready for school | 12.3 (31,454) | 12.2 (31,377) | 16.9 (77) |
|  | Missing | 0.9 (2,323) | 0.9 (2,321) | <0.9 (<5) |
|  | Advanced literacy |  |  |  |
|  | Ready for school | 75.7 (194,227) | 75.7 (193,918) | 67.8 (309) |
|  | Not ready for school | 21.8 (56,047) | 21.8 (55,912) | 29.6 (135) |
|  | Missing | 2.5 (6,355) | 2.5 (6,343) | 2.6 (12) |
|  | Basic numeracy |  |  |  |
|  | Ready for school | 84.2 (216,121) | 84.2 (215,765) | 78.1 (356) |
|  | Not ready for school | 15.2 (39,122) | 15.2 (39,026) | 21.1 (96) |
|  | Missing | 0.5 (1,386) | 0.5 (1,382) | <1.1 (5) |
| Note: CHD, Congenital heart disease. There were no subdomains within the domain of communication skills and general knowledge, which was therefore not included in this analysis. Cells with counts <5 were suppressed in accordance with confidentiality requirements. | | | | |

| **Table F.** Four-way decomposition of the association between maternal CHD and child developmental vulnerability with preterm birth as mediator and effect modifier. | | |
| --- | --- | --- |
| **Parameters** | **Excess Relative Risk**  (95% CI) | **Attributable Proportion**  % of Total Excess Relative Risk |
| Controlled Direct Effect (CDE) | 0.25 (0.06, 0.44) | 91.5% |
| Reference Interaction (INTref) | -0.00 (-0.01, 0.00) | -0.2% |
| Mediated Interaction (INTmed) | 0.00 (-0.03, 0.03) | 0.5% |
| Pure Indirect Effect (PIE) | 0.02 (0.01, 0.03) | 8.2% |
| Total Excess Relative Risk (TERERI) | 0.27 (0.08, 0.46) | 100% |
| Total Effect Risk Ratio (TERIRA) | 1.27 (1.08, 1.46) |  |
| Note: CI, Confidence interval; CHD, Congenital heart disease.  TERERI = TERIRA – 1 = CDE + INTref + INTmed + PIE  All effect parameters were estimated at the mean level of the covariates: child’s sex, birth year, and age at EDI completion, and maternal age at delivery, parity, country of birth, marital status, neighbourhood income quintiles, pre-existing psychiatric disorders, and pre-gestational diabetes.  A Poisson model was specified for the outcome and a logistic model for the mediator. The 95% CIs were obtained through bootstrapping, with 1000 replications. | | |

| **Table G.** Effect modification by maternal pregnancy complications and neighbourhood income quintiles on the association between maternal CHD and early childhood developmental vulnerability, additive scale. | | | | |
| --- | --- | --- | --- | --- |
|  | Additive interaction  (Common reference group) | | | |
| Subgroups | No. of children | Number of events (%) | Adjusted***** RR  (95% CI) | RERI (95% CI);  AP |
| **Ischemic placental disease (IPD)** | | | | |
| CHD-/IPD- | 242,655 | 39,620 (16.3) | 1.00 (Ref.) | -0.49 (-1.16, 0.18);  -42.2% |
| CHD+/IPD- | 424 | 107 (25.2) | 1.32 (1.11, 1.52) |  |
| CHD-/IPD+ | 13,518 | 3,001 (22.2) | 1.32 (1.28, 1.37) |  |
| CHD+/IPD+ | 32 | <10 | 1.15 (0.51, 1.79) |  |
| **Gestational diabetes (GDM)** | | | | |
| CHD-/GDM- | 237,590 | 38,957 (16.4) | 1.00 (Ref.) | 0.90 (-0.03, 1.82); 38.9% |
| CHD+/GDM- | 425 | 101 (23.8) | 1.22 (1.03, 1.42) |  |
| CHD-/GDM+ | 18,583 | 3,664 (19.7) | 1.19 (1.15, 1.23) |  |
| CHD+/GDM+ | 31 | 14 (45.2) | 2.31 (1.40, 3.22) |  |
| **Neighbourhood income quintiles (Q)** | | | | |
| CHD-/Q5 | 41,968 | 5,197 (12.4) | 1.00 (Ref.) | 0.21 (-0.59, 1.00); 12.7% |
| CHD+/Q5 | 74 | 14 (18.9) | 1.32 (0.76, 1.88) |  |
| CHD-/Q4 | 51,821 | 7,185 (13.9) | 1.09 (1.06, 1.13) |  |
| CHD+/Q4 | 89 | 20 (22.5) | 1.62 (1.06, 2.18) |  |
| CHD-/Q3 | 53,382 | 8,183 (15.3) | 1.17 (1.13, 1.20) | -0.20 (-0.98, 0.58);  -15.5% |
| CHD+/Q3 | 82 | 16 (19.5) | 1.29 (0.75, 1.82) |  |
| CHD-/Q2 | 54,981 | 9,913 (18.0) | 1.29 (1.25, 1.33) | -0.20 (-0.94, 0.54); -14.2% |
| CHD+/Q2 | 97 | 22 (22.7) | 1.41 (0.92, 1.89) |  |
| CHD-/Q1 | 54,021 | 12,143 (22.5) | 1.48 (1.43, 1.52) | 0.25 (-0.48, 0.99); 12.3% |
| CHD+/Q1 | 114 | 43 (37.7) | 2.05 (1.57, 2.53) |  |
| Note: AP, Attributable proportion; CHD, Congenital heart disease; CI, Confidence interval; GDM, Gestational diabetes mellitus; IPD, Ischemic placental disease; Q, Quintile; RERI, Relative excess risk due to interaction; RR, Risk ratio.  *****Adjusted for child’s sex, birth year, and age at EDI completion, and maternal age at delivery, parity, country of birth, marital status, neighborhood income quintiles, pre-existing psychiatric disorders, and pre-gestational diabetes. | | | | |

| **Table H.** Risk ratios from multiple imputation analysis for the association between maternal CHD and child’s overall developmental vulnerability, with additional adjustment for maternal BMI. (N=222,275) | | | | | |
| --- | --- | --- | --- | --- | --- |
| **Maternal CHD** | **Total** | **Children with vulnerability**  % (n) | **Model 1** | **Model 2** | **Model 3** |
|  |  |  | RR (95% CI) | RR (95% CI) | RR (95% CI) |
| No | 221,846 | 17.0 (37,675) | 1.00 (Ref.) | 1.00 (Ref.) | 1.00 (Ref.) |
| Yes | 429 | 26.6 (114) | 1.56 (1.34, 1.83) | 1.33 (1.15, 1.54) | 1.31 (1.13, 1.52) |
| Note: BMI, Body mass index; CHD, Congenital heart disease; CI, Confidence interval; RR, Risk ratio.  This analysis was restricted to children born from 2000 onward since data on maternal BMI before that calendar year was not available. The RRs and 95% CIs represent pooled estimates from 35 imputed datasets.  Model 1: Unadjusted  Model 2: Adjusted for child’s sex, birth year, and age at EDI completion, and maternal age at delivery, parity, country of birth, marital status, neighbourhood income quintiles, pre-existing psychiatric disorders, and pre-gestational diabetes.  Model 3: Model 2 + maternal BMI | | | | | |

| **Table I.** Sensitivity analysis of the association between maternal CHD and child’s overall developmental vulnerability, after excluding child CHD and major congenital malformations. (N=249,141) | | | | |
| --- | --- | --- | --- | --- |
| **Maternal CHD** | **Total** | **Children with vulnerability**  % (n) | **Crude** | **Adjusted*** |
|  |  |  | RR (95% CI) | RR (95% CI) |
| No | 248,710 | 16.4 (40,788) | 1.00 (Ref.) | 1.00 (Ref.) |
| Yes | 431 | 23.9 (103) | 1.46 (1.23, 1.73) | 1.24 (1.06, 1.45) |
| Note: CHD, Congenital heart disease; CI, Confidence interval; RR, Risk ratio.  *Adjusted for child’s sex, birth year, and age at EDI completion, and maternal age at delivery, parity, country of birth, marital status, neighbourhood income quintiles, pre-existing psychiatric disorders, and pre-gestational diabetes. | | | | |

| **Table J.** Risk ratios for the association between maternal CHD severity and child’s overall developmental vulnerability. | | | | |
| --- | --- | --- | --- | --- |
| **Maternal CHD severity** | **Total** | **Children with vulnerability**  % (n) | **Crude** | **Adjusted*** |
|  |  |  | RR (95% CI) | RR (95% CI) |
| No CHD | 256,193 | 16.4 (42,623) | 1.00 (Ref.) | 1.00 (Ref.) |
| Mild | 299 | 26.2 (79) | 1.59 (1.31, 1.92) | 1.19 (1.00, 1.42) |
| Moderate | 97 | 20.6 (20) | 1.24 (0.84, 1.83) | 1.15 (0.78, 1.69) |
| Severe | 40 | 35.0 (14) | 2.10 (1.38, 3.21) | 1.98 (1.31, 3.00) |
| Note: CHD, Congenital heart disease; CI, Confidence interval; RR, Risk ratio.  *Adjusted for child’s sex, birth year, and age at EDI completion, and maternal age at delivery, parity, country of birth, marital status, neighbourhood income quintiles, pre-existing psychiatric disorders, and pre-gestational diabetes.  Ratio of the RRs for severe and mild CHD: 1.66 (1.06, 2.61) | | | | |

| **Table K.** Risk ratios for the associations between maternal CHD and child’s overall developmental vulnerability, stratified by ICD-9 versus ICD-10 coding systems. | | | | |
| --- | --- | --- | --- | --- |
|  | **ICD-10 definition of maternal CHD** | | | |
| **Maternal**  **CHD** | **Total** | **Children with vulnerability**  % (n) | **Crude** | **Adjusted*** |
|  |  |  |  |  |
|  |  |  | RR (95% CI) | RR (95% CI) |
| No | 256,512 | 16.6 (42,708) | 1.00 (Ref.) | 1.00 (Ref.) |
| Yes | 117 | 23.9 (28) | 1.44 (1.04, 1.99) | 1.29 (0.96, 1.75) |
|  | **ICD-9 definition of maternal CHD** | | | |
| No | 256,200 | 16.6 (42,629) | 1.00 (Ref.) | 1.00 (Ref.) |
| Yes | 429 | 24.9 (107) | 1.50 (1.27, 1.77) | 1.25 (1.07, 1.45) |
| Note: CHD, Congenital heart disease; CI, Confidence interval; ICD, International Classification of Disease; RR, Risk ratio.  *Adjusted for child’s sex, birth year, and age at EDI completion, and maternal age at delivery, parity, country of birth, marital status, neighbourhood income quintiles, pre-existing psychiatric disorders, and pre-gestational diabetes. | | | | |

| **Table L.** Risk ratios for the association between maternal CHD and child’s overall developmental vulnerability, with additional adjustment for maternal use of cardiac medications in pregnancy. | | | | | |
| --- | --- | --- | --- | --- | --- |
| **Maternal CHD** | **Total** | **Children with vulnerability**  % (n) | **Model 1** | **Model 2** | **Model 3** |
|  |  |  | RR (95% CI) | RR (95% CI) | RR (95% CI) |
| No | 256,193 | 16.6 (42,621) | 1.00 (Ref.) | 1.00 (Ref.) | 1.00 (Ref.) |
| Yes | 456 | 25.2 (115) | 1.52 (1.29, 1.78) | 1.27 (1.10, 1.47) | 1.27 (1.10, 1.48) |
| Note: CHD, Congenital heart disease; CI, Confidence interval; RR, Risk ratio.  Model 1: Unadjusted  Model 2: Adjusted for child’s sex, birth year, and age at EDI completion, and maternal age at delivery, parity, country of birth, marital status, neighbourhood income quintiles, pre-existing psychiatric disorders, and pre-gestational diabetes.  Model 3: Model 2 + maternal use of cardiac medications | | | | | |

| **Table M.** Sensitivity analysis of the association between maternal CHD and child’s overall developmental vulnerability, after excluding children of foreign-born mothers. (N=185,357) | | | | |
| --- | --- | --- | --- | --- |
| **Maternal CHD** | **Total** | **Children with vulnerability**  % (n) | **Crude** | **Adjusted*** |
|  |  |  | RR (95% CI) | RR (95% CI) |
| No | 184,961 | 16.4 (30,390) | 1.00 (Ref.) | 1.00 (Ref.) |
| Yes | 396 | 26.8 (106) | 1.63 (1.38, 1.82) | 1.30 (1.12, 1.52) |
| Note: CHD, Congenital heart disease; CI, Confidence interval; RR, Risk ratio.  *Adjusted for child’s sex, birth year, and age at EDI completion, and maternal age at delivery, parity, marital status, neighbourhood income quintiles, pre-existing psychiatric disorders, and pre-gestational diabetes. | | | | |

| **Table N.** Sensitivity analysis of the association between maternal CHD and child’s overall developmental vulnerability, after excluding children participating in the pilot or Wave 1 survey. (N=222,275) | | | | |
| --- | --- | --- | --- | --- |
| **Maternal CHD** | **Total** | **Children with vulnerability**  % (n) | **Crude** | **Adjusted*** |
|  |  |  | RR (95% CI) | RR (95% CI) |
| No | 184,171 | 17.0 (37,675) | 1.00 (Ref.) | 1.00 (Ref.) |
| Yes | 315 | 26.6 (104) | 1.56 (1.34, 1.83) | 1.32 (1.14, 1.52) |
| Note: CHD, Congenital heart disease; CI, Confidence interval; RR, Risk ratio.  *Adjusted for child’s sex, birth year, and age at EDI completion, and maternal age at delivery, parity, marital status, neighbourhood income quintiles, pre-existing psychiatric disorders, and pre-gestational diabetes. | | | | |

| **Table O.** Sensitivity analysis of the association between maternal CHD and child’s overall developmental vulnerability, excluding outpatient CHD diagnoses without a cardiology-specific claims. (N=255,039) | | | | |
| --- | --- | --- | --- | --- |
| **Maternal CHD** | **Total** | **Children with vulnerability**  % (n) | **Crude** | **Adjusted*** |
|  |  |  | RR (95% CI) | RR (95% CI) |
| No | 254,583 | 16.6 (42,257) | 1.00 (Ref.) | 1.00 (Ref.) |
| Yes | 456 | 25.2 (25.2) | 1.52 (1.30, 1.78) | 1.28 (1.11, 1.49) |
| Note: CHD, Congenital heart disease; CI, Confidence interval; RR, Risk ratio.  *Adjusted for child’s sex, birth year, and age at EDI completion, and maternal age at delivery, parity, country of birth, marital status, neighbourhood income quintiles, pre-existing psychiatric disorders, and pre-gestational diabetes. | | | | |

| **Table P.** Sensitivity analysis of the association between maternal CHD and child’s overall developmental vulnerability, excluding potential Patent Foramen Ovale cases. (N=256,533) | | | | |
| --- | --- | --- | --- | --- |
| **Maternal CHD** | **Total** | **Children with vulnerability**  % (n) | **Crude** | **Adjusted*** |
|  |  |  | RR (95% CI) | RR (95% CI) |
| No | 256,173 | 16.6 (42,621) | 1.00 (Ref.) | 1.00 (Ref.) |
| Yes | 360 | 25.3 (91) | 1.52 (1.27, 1.82) | 1.29 (1.09, 1.52) |
| Note: CHD, Congenital heart disease; CI, Confidence interval; RR, Risk ratio.  *Adjusted for child’s sex, birth year, and age at EDI completion, and maternal age at delivery, parity, country of birth, marital status, neighbourhood income quintiles, pre-existing psychiatric disorders, and pre-gestational diabetes. | | | | |

| **Table Q.** Sensitivity analysis of the association between maternal CHD and child’s overall developmental vulnerability, with death before age-5 treated as developmental vulnerability. (N=258,142) | | | | |
| --- | --- | --- | --- | --- |
| **Maternal CHD** | **Total** | **Children with vulnerability**  % (n) | **Crude** | **Adjusted*** |
|  |  |  | RR (95% CI) | RR (95% CI) |
| No | 257,680 | 17.1 (44,142) | 1.00 (Ref.) | 1.00 (Ref.) |
| Yes | 462 | 26.2 (121) | 1.53 (1.31, 1.78) | 1.28 (1.10, 1.47) |
| Note: CHD, Congenital heart disease; CI, Confidence interval; RR, Risk ratio.  *Adjusted for child’s sex, birth year, and age at death or EDI completion, and maternal age at delivery, parity, country of birth, marital status, neighbourhood income quintiles, pre-existing psychiatric disorders, and pre-gestational diabetes. | | | | |
